# Impacts of COVID‐19 on global poverty, food security, and diets: Insights from global model scenario analysis

**DOI:** 10.1111/agec.12624

**Published:** 2021-04-08

**Authors:** David Laborde, Will Martin, Rob Vos

**Affiliations:** ^1^ Markets, Trade and Institutions Division at IFPRI International Food Policy Research Institute Washington District of Columbia USA

**Keywords:** CGE analysis, COVID‐19, dietary change, food security, poverty

## Abstract

This study assesses the impact of coronavirus disease 2019 (COVID‐19) on poverty, food insecurity, and diets, accounting for the complex links between the crisis and the incomes and living costs of vulnerable households. Key elements are impacts on labor supply, effects of social distancing, shifts in demand from services involving close contact, increases in the cost of logistics in food and other supply chains, and reductions in savings and investment. These are examined using IFPRI's global general equilibrium model linked to epidemiological and household models. The simulations suggest that the global recession caused by COVID‐19 will be much deeper than that of the 2008–2009 financial crisis. The increases in poverty are concentrated in South Asia and sub‐Saharan Africa with impacts harder in urban areas than in rural. The COVID‐19‐related lockdown measures explain most of the fall in output, whereas declines in savings soften the adverse impacts on food consumption. Almost 150 million people are projected to fall into extreme poverty and food insecurity. Decomposition of the results shows that approaches assuming uniform income shocks would underestimate the impact by as much as one‐third, emphasizing the need for the more refined approach of this study.

## 1 INTRODUCTION

Global cases of coronavirus disease 2019 (COVID‐19) worldwide have grown exponentially since February 2020, despite progress on managing this pandemic in some countries, with worldwide daily reported new cases rising from around 500 in late February to almost 600,000 by November, with the threat of further increases during the northern‐hemisphere winter. The epicenter of the pandemic shifted from China to Europe and then to the United States and Latin America, with the disease resurgent in the northern autumn. COVID‐19 is now also spreading rapidly in low‐ and middle‐income countries in Africa and Asia, many of which lack robust health systems or strong social safety nets that can soften the pandemic's public health and economic impacts.

More than half of the world population has been, still is, or is again under some form of social distancing regime designed to contain the health crisis. Business activity has fallen sharply because of a combination of policy action and personal responses designed to reduce risk of contracting the virus, with personal action probably more important than policy in reducing economic activity (Goolsbee & Syverson, 2020). The International Labour Organization estimates that during the first three quarters of 2020, the number of working hours worldwide declined by 17% relative to that in the last quarter of 2019; a drop equivalent to a loss of almost 500 million full‐time jobs (ILO, 2020a). Governments in Europe, the United States, and other high‐income countries have taken unprecedented fiscal and monetary stimulus measures to compensate for the income losses of businesses and workers and contain an inevitable economic crisis. But the relief responses of low‐ and middle‐income countries have been more limited.

COVID‐19 poses a serious threat to global food security through various transmission mechanisms (Laborde, Martin, Swinnen, & Vos, 2020). From what is currently known, the worst of these threats is the global economic recession causing many to lose income and leaving many vulnerable people unable to afford the food they need. Income declines not only reduce demand for food but also induce shifts in the mix of products consumed, notably resulting in less consumption of more nutrient‐rich foods (like fruits, vegetables, and animal‐sourced foods) and relatively more of calorie‐rich foods (like basic grains and sugar). Other threats arise from disruptions in agricultural input markets, farm production, marketing, and distribution of food caused by the need for social distancing to combat the global health crisis.

As COVID‐19 and its economic fallout spread in the poorest parts of the world, more people have become poor and food insecure. Although some context‐specific estimates of the impacts of these shocks on poverty and food insecurity are available, it will be years before comprehensive and comparative survey‐based information on these impacts become available. A key contribution of this paper is to assess these impacts using an integrated global modeling framework that includes national and household models. In a new scenario analysis, presented in this study, we estimate that globally, absent adequate responses in poorer nations, close to 150 million more people could fall into extreme poverty (measured against the PPP$1.90 poverty line) in 2020—an increase of 20% from prepandemic levels. This, in turn, would drive up food insecurity.

Assessing the poverty impact of COVID‐19 is no trivial matter, however. This is so not only because the crisis is still unfolding and available information of its precise socioeconomic consequences is incomplete, but also because the channels of influence are multiple and interconnected globally. Although several analyses of the poverty impacts have used simple tools provided by the World Bank's PovcalNet website^1^ and assumed uniform shifts in the distribution of income per country to provide estimates of the impacts on poverty (see, for example, the studies by the World Bank in Mahler, Lakner, Aguilar, & Wu, 2020 and World Bank, 2020b; and that of UN‐WIDER by Sumner, Hoy, & Ortiz‐Juarez, 2020), we are concerned that this assumption fails to account for the complexity of the channels of effect and may substantially underestimate the impacts of the pandemic. Our methodology allows to account for the disproportionate impact of the pandemic on the poor (Swinnen, 2020), something neglected in analyses using uniform shifts in all incomes. Results from a range of studies examining the impacts of COVID‐19 on GDP and on poverty are presented in Online Appendix A.5. This shows that estimates of the severity of the impact increased dramatically after March 2020. The results of this study fall within the range of other estimates.

In this paper, we use information on the nature of the shocks to income, the structure of the global economy, and linked household models to provide more detailed estimates of the likely implications for income distribution, poverty, and the food security of vulnerable families. The next section of the paper looks at the transmission channels from COVID‐19 to poverty and food security. The third examines our modeling framework, including the MIRAGRODEP global computable general equilibrium (CGE) model and the POVANA framework. The fourth section presents the key assumptions of the COVID‐19 scenario used in the analysis, whereas the fifth presents key results from the analysis and identifies the main transmission channels of the global macroeconomic and poverty impacts. A sixth section provides an update of the reference scenario to illustrate the sensitivity of the results to changes in key assumptions and to validate those assumptions against the most recent available evidence about observed impacts of the pandemic. The final section concludes.

## 2 TRANSMISSION CHANNELS OF COVID‐19′S IMPACT ON POVERTY, FOOD SECURITY, AND NUTRITION

COVID‐19 has smaller direct impacts on agricultural production than many other pandemics. The 1918 “Spanish Flu” pandemic, for example, caused substantial losses in farm output because of high morbidity and mortality among working‐age males (Schultz, 1964). Some other pandemics, such as Swine flu and Avian flu, have directly reduced agricultural production. By contrast, COVID‐19 involves a relatively short period of sickness for most of its victims, has its highest mortality rates among older people, many of whom have left the formal workforce, and does not directly affect crops or livestock. However, it does have substantial impacts on agriculture and food security, generally through less direct channels of influence. Therefore, it is useful to begin the discussion by laying out the channels through which COVID‐19 affects food markets and food security. We then turn to the modeling framework that we use to evaluate these impacts.

The main channels of effect between the COVID‐19 pandemic and food security are:

- income losses and demand shocks;
- food supply chain disruptions;
- consumer responses, such as hoarding, food waste, and dietary shifts;
- policy responses: hoarding at country level (food export bans) and fiscal stimulus.

*Income losses* play an important role in reducing food security during the COVID‐19 pandemic. We know from the work of Amartya Sen (1981) that food insecurity and even famines frequently are not associated with physical shortages of food. What matters more is people's ability to access food. Some of the current income declines are direct consequences of the disease, such as working time lost due to the disease; whereas others are policy responses designed to reduce the rate of disease transmission. It appears that the most important are individual responses as people try to avoid situations where they are likely to catch (or transmit) the disease (Goolsbee & Syverson, 2020). Because individuals consider primarily their own risk of infection, some degree of coordinated distancing is appropriate to reduce the externalities imposed on others and particularly the loss of life associated with the pandemic. These social distancing policies range from simple measures such as encouraging wearing of masks and frequent handwashing, through more intrusive policies such as restricting activities with high transmission risk, to strict lockdown requirements.

The income losses resulting from these actions are primarily outside the food system as food‐related activities have generally been designated “essential” activities exempt from being locked down, except for some restaurants and other food‐away from home outlets. Hence, most of the direct income losses are outside the agri‐food system. Unskilled workers in nonessential activities are at greatest risk of falling into unemployment because they generally do not have the telecommuting options that have greatly reduced the impact of this pandemic on overall economic activity and employment.

*Food supply chain disruptions* caused by COVID‐19 are also affecting food security. Staple food production in high‐income countries has been relatively little affected, whereas labor‐intensive activities in some markets and processing activities have been strongly affected by disease outbreaks. Another key point of breakdown has been in processing of some agricultural products—and particularly production of meat—where low temperatures and proximity of workers can result in very high rates of disease transmission. Other disruptions to food supply chains have come from restriction on the movement of workers, the dramatic reduction in international air travel, and slowdowns in the administrative approvals for food trade. At the consumer end, restaurant services have been particularly hard hit both by lockdown policies and by consumer risk aversion.

Most *consumer responses* have been consequences of the COVID shocks, but some have injected additional volatility into the system. Uncertainty about the impact of the pandemic on availability of some foods has added volatility to food demand as consumers have sought to stockpile food items, such as meat and dairy products. Another early feature of adjustment to the pandemic was increased food loss as suppliers struggled to adjust their product mix in response to shifts in final sales away from food services to consumption at home. A third feature of adjustment appears to have been a run down in financial assets as affected households seek to reduce the impact of income losses on their access to food. In one carefully studied case, Abate, de Brauw, and Hirvonen (2020) found that only a small fraction of Ethiopian households appear to have enough savings to cover more than a month's food needs. The same study tracking households during the COVID‐19 outbreak also finds that income losses and food price changes appear to have changed demand for food, with declines in consumption of nutrient‐rich products like legumes, vegetables, and dairy.

*Policy responses* to the pandemic also play a major role in the outcome. Although economies would likely have had substantial reductions in economic activity as people sought to avoid catching (and/or transmitting) the disease, lockdown policies appear to have increased the adverse short‐run impact on output, while—where properly implemented—reducing the rate of transmission and potentially allowing a swifter recovery. In some cases, this has had a high payoff, by sharply reducing the impact of the disease, while, in other cases, such as the United States, the opportunity to reduce the incidence of the disease to low levels in the first round was missed. Even when containment policies were initially successful, frequent resurgences of the disease suggest that the economic impacts are likely to last until effective treatments and/or vaccines are widely available.

Fiscal and monetary stimulus appears to have had a substantial impact on output levels in many of the higher income countries, with initial fiscal stimulus of around 11% of GDP in the United States and substantial stimulus packages in many other high‐income countries.^2^ Although fiscal stimulus packages have been announced in many developing countries, these generally appear to be much smaller as a share of GDP than those in the higher income countries. Expansion of social protection programs has been an important element in the response with 212 countries, mostly in the developing world, introducing almost 1200 measures by September 2020.^3^ About half of the social assistance measures were cash based, with most being short term in duration. In developing countries, the size and duration of such responses seems to be highly variable. As little is known so far about the precise allocation of those resources across households, we do not account for the social protection measures taken by developing countries in the scenario analysis presented below. Our focus is rather on assessing the direct impact of the crisis on poverty in the absence of such social protection measures.

Many countries implemented restrictions on food exports early in the crisis designed to avoid increases in domestic food prices (Martin & Glauber, 2020). Fortunately, however, these restrictions did not set off an upward price spiral of the type seen in 2007–2008 (Anderson, Ivanic, & Martin, 2014). Although 22 countries had announced or imposed food export restrictions, affecting around 5% of calories embedded in traded food, early in the crisis, all but one had been eliminated by the end of September.^4^

## 3 THE MODELING FRAMEWORK

We use a global modeling framework to assess the potential impacts of the COVID‐19 crisis on global poverty and food security. Specifically, we combine two economic modeling frameworks: IFPRI's global CGE model, MIRAGRODEP,^5^ and the POVANA household dataset and model. This framework has been used previously to study the impact of a macroeconomic slowdown on global poverty in Laborde and Martin (2018). The main differences between the current work and the previous study are twofold. First, the Laborde–Martin study looks at a change in economic growth projections for 2015–2030 and compared poverty outcomes in 2030, using the dynamic version of the CGE and projecting household surveys until 2030.

In the current exercise, we focus on single‐year (2020) scenario results under a range of assumptions about short‐term impacts of COVID‐19, as explained further below. Second, in Laborde and Martin (2018), alternative IMF projections for global growth are regenerated by imposing commensurate changes in total factor productivity on the corresponding MIRAGRODEP parameter values. In contrast, in the current exercise, the factors underlying the socioeconomic impacts of COVID‐19, such as health impacts, social distancing, restrictions on (labor) mobility, international transport, and the closure of some business activities, are translated into MIRAGRODEP's model terms to simulate endogenously the impacts on economic growth, incomes, employment, consumption, prices, trade, and ultimately, poverty.

**FIGURE 1:**
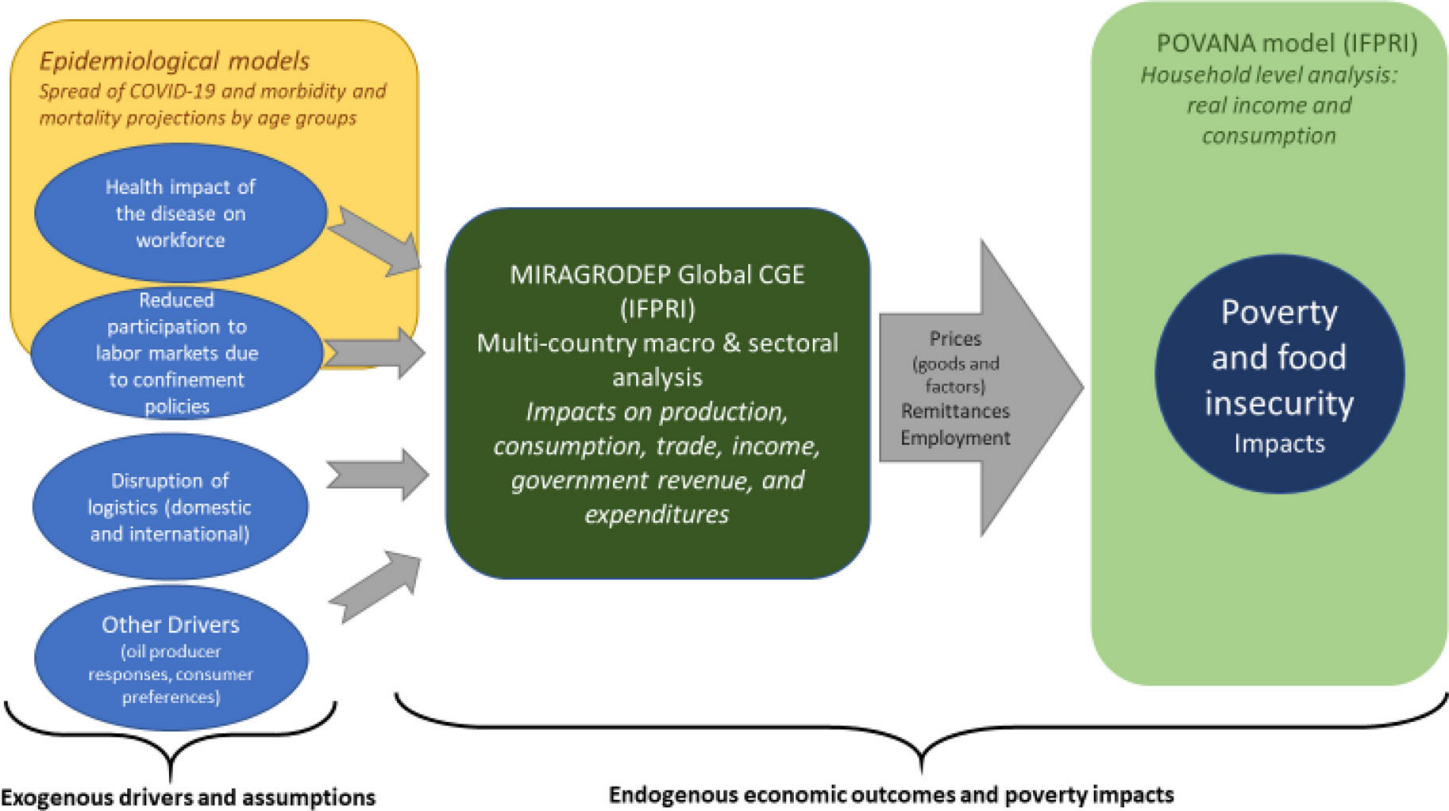
Implementation of the Covid‐19 scenarios [Color figure can be viewed at wileyonlinelibrary.com] *Source*: Authors’ depiction.

The two modeling frameworks are linked in top‐down fashion; that is, the relevant results of the CGE model‐based scenario analysis are introduced, along with the direct impacts of the pandemic on households, as shocks to the household survey model to assess poverty outcomes. In addition, the health impacts of the disease on labor supply and productivity are linked to outcomes from epidemiological models. This process is summarized in Figure 1.

The main technical features of the MIRAGRODEP and POVANA models and their linkages are summarized in Online Appendix A.1. For the present analysis, we assume in the MIRAGRODEP model that unskilled workers are harder hit than skilled workers by social distancing measures, as skilled workers are more likely able to continue work from home. We assume further that producers have very little ability to change the capital–labor utilization ratio within a single year. Governments in high‐income countries are assumed to have put in place economic stimulus measures (see below under scenario assumptions), while—for the present analysis—those of poorer countries are assumed to have limited ability to borrow to provide such substantial stimulus, and so maintain the public deficit/surplus to GDP constant.

The POVANA household model uses data on the full income distribution for around 300,000 households.^6^ Having this detail avoids having to make ex‐ante or ad‐hoc assumptions about how the economic shocks caused by COVID‐19 change the distribution of income in any given country. In our approach, real incomes of households change endogenously with the simulated changes in the full vector of changes in employment; changes in prices of goods, services, and factors (including wages); and other income determinants (productivity). Changes in poverty levels are calculated by comparing the poverty rates before and after the changes in household incomes.

**FIGURE 2:**
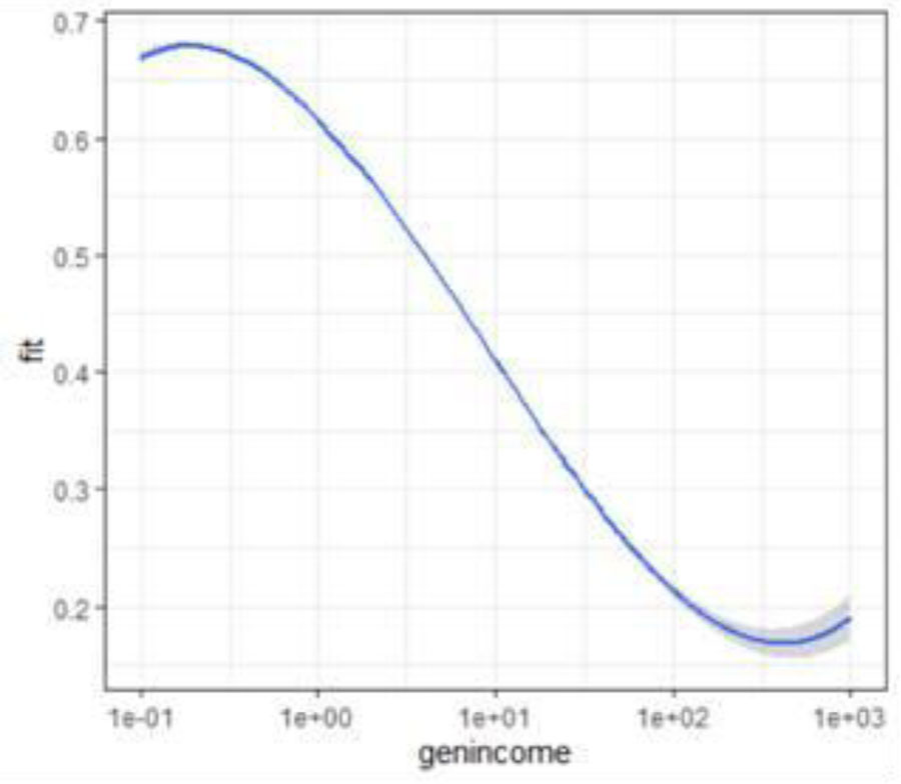
Engel's law: Declining food expenditure shares with rising incomes [Color figure can be viewed at wileyonlinelibrary.com] *Source*: POVANA database. Authors’ computation. *Note*: The blue line represents the estimated share of food consumption in total expenditures estimated through a polynomial of degree 3 on the log of individual income household, normalized by their own country's poverty line.

Finally, the POVANA database provides information about household consumption patterns. This also allows identification of the impacts of economic shocks (like the consequences of COVID‐19) on the costs of goods consumed by the household, and particularly on the costs of food consumed. Income losses and food price shocks will disproportionately hurt poor people's food security, because they spend most of their income on food: as much as 70%. Rich people spend only a small share—perhaps around 15%—of their incomes on food (Figure 2). The most immediate threat of COVID‐19 to food security arises from reductions in the incomes of poor and vulnerable people. Some of these losses arise from income losses in agriculture, but a much larger share of these income losses arises from disruption to nonagricultural income sources.

## 4 THE COVID‐19 SCENARIO

We model a range of impacts of the COVID‐19 pandemic. Beyond the direct effects of the disease on the ability to work, income losses arise from people's desire to avoid catching the disease and their altruistic concerns to avoid infecting other people, and from policy responses designed to reduce the adverse externalities associated with an unmitigated pandemic. No global economy‐wide model incorporating these features is available to fully assess these potential impacts and behavioral changes. Many of the changes in behavior and in the functioning of economies are not yet fully understood and their impacts on economic activity were still not fully known when preparing this scenario analysis. It is also difficult to rely on experience from past events, because no events like the COVID‐19 pandemic have occurred on this scale in today's globalized world. Therefore, we have had to make several assumptions about the responses of economic agents to this unprecedented situation.

In crafting the scenarios used here, we have based our choices on earlier work, such as the analysis we undertook in March 2020,^7^ when we looked at the differential impacts on productivity and trade costs for a 1% global economic slowdown during 2020. Before looking at the specific scenario assumptions, it is important to keep in mind that the model operates on an annual time step and the impacts of any shock are calculated as the average impact for the year. Therefore, a disruption lasting 10 days is associated with a 10/365 impact and a price shock, for example, such as the decline in oil prices, must be calibrated on the shift in annual average prices and not on the “peak” value.

We distinguish four drivers of COVID‐19 impacts: domestic supply disruptions, global market disruptions, household behavioral responses, and policy responses.

### 4.1 Domestic supply disruptions

#### 4.1.1 Disruptions in labor markets

We consider two broad impacts on labor markets. The first is the direct impact of mortality and morbidity on labor supply. The second is the impacts on labor supply of social distancing actions undertaken to reduce transmission of the disease. The first impact is linked to the direct impact of the disease. For our reference scenario, we use estimates provided by Imperial College London for each country (Walker et al., 2020).^8^ Specifically, we use the “Social distancing of the whole population” scenario for all countries. As their online materials do not provide results by age cohorts, we reestimated those, following a procedure explained in Online Appendix A.3. We note that this direct effect is generally quite small due compared to the next type of disruption.

Social distancing results in some willing workers become unable to sell their labor. In our reference scenario, we use the “social‐distancing” parameter from the Imperial College estimates as a base value, and assume that 12 weeks of confinement is imposed in each country, except in African countries, for which we limit it to 8 weeks, due to the more limited ability of poor populations to manage long periods of economic disruption; lower population densities than in South Asian countries; the younger average age of people in the region and the consequent more relaxed implementation of confinement policies. These assumptions result in reductions in labor supply of 23% in most countries or 15% in Africa. We consider that one‐third of skilled workers impacted by social distancing can continue working through telecommuting. This crude estimate is based on the ILO's early review of the impact of COVID‐19 on jobs of April 2020 (ILO, 2020b) and Dingel and Neiman (2020).^9^

#### 4.1.2 Disruptions in specific value chains

Although agriculture and food sectors have been identified as essential in most countries, we also assume some supply disruption caused by reduced labor mobility (e.g., for seasonal migrant labor) and further, that perishable farm products suffer greater postharvest losses due to logistics problems and demand fallout. An increase in postharvest losses of perishable products (fruits, vegetables, meat, and dairy) of five points is included. Although this estimate is conjectural, anecdotal evidence suggests that losses have been substantial in some cases and minimal in others making an average loss of 5% seem a reasonable guesstimate for the present purpose of analysis

Total factor productivity in transportation is assumed to decline by 5% to capture losses of logistical efficiency. This number is extrapolated based on anecdotal evidence ranging from monitoring of GPS tracking devices on truck fleets in the United States (see the work of ATRI)^10^ and from recent surveys conducted in West Africa.^11^ While crude, this estimate provides at least a reasoned estimate of the extent of disruption to transportation sectors, especially in developing countries.

Because both autonomous social distancing (driven by fear of catching the disease) and lockdown policies designed to reduce externalities tend to reduce activity in high‐contact services such as restaurants, travel, bars, and gyms, we introduce a “shadow tax”^12^ of 25% for both final and intermediate consumption of these services. This reduces the demand for these services, *ceteris paribus*, by about one‐third on average.

### 4.2 Global market disruptions

To capture the effects of the “oil war” between Saudi Arabia and Russia in late 2019 and early 2020 but predating COVID‐19, we introduce an exogenous expansion of the supply of oil. The combined effect of this larger supply of oil and the lower demand caused by the COVID‐19 crisis induces a drop in global real energy prices by 25% for crude oil and natural gas and 17% for refined oil and gas products.^13^

The containment measures cause bottlenecks and delays in international freight and transport. In terms of the model parameters, this assumption has been translated into an increase in the average cost of international freight by 3%, not considering any feedback on energy prices. We calibrate these numbers to capture the increased time required to trade, because of logistical delays in harbors and at airports caused by new regulations, lack of inspectors, and other frictions associated with the pandemic. These lost days are converted into ad‐valorem equivalents using a procedure developed by Hummels and Schaur (2013).

### 4.3 Household and business responses

We assume that private sector agents and businesses reduce their savings as a coping mechanism to compensate for the adverse impact of the pandemic on current incomes. In the global CGE model, the savings reduction is defined for each country/region subject to two constraints: first, to the extent they can, private sector agents try to limit their welfare loss to 5% of initial income, but, second, they cannot cut their savings rates by more than 6% of initial income and cannot let their savings become negative. These boundaries were chosen based on changes in gross saving rates observed in previous crises. For instance, in the United States, between 2006 and 2009, the gross savings rate fell from 18.0% to 15.1%, whereas the world average declined from 26.6% to 24.1%.^14^

It should be noted that MIRAGRODEP cannot fully capture the differences in savings behavior across economic agents. Typically, in contrast to the above, household savings tend to increase during recessions, which Keynes characterized as the “paradox of thrift” (Keynes, 1936). Although poor households may be unable to save and may even need to dispose of assets to survive, more affluent households try to save more in uncertain times, reducing consumption and thereby deepening the recession. In the United States, for instance, COVID‐19 substantially limited consumption spending, leading the personal savings rate (as a share of disposable income) to increase from around 7% in early 2020 to 32% in April to taper off to 23% in May of the same year.^15^ Overall savings appear to be down, however, with the fall in corporate savings being larger than the increase in household savings, as happened during the Great Recession of 2008–2009,^16^ and, as a result, investment decline as well. In MIRAGRODEP, the corporate sector is included with the household sector, so we assume that the expected impact of COVID‐19 on corporate savings predominates the aggregate impact, with overall savings declining.

The composition of food demand will also change during the recession. Households are expected to reduce demand for fresh products (such as fruits, vegetables, meats, and fish). This food demand shift is endogenous to income and price shifts in the model. The simulated impacts shown further below could underestimate the true effects, because we do not account for changes in consumer perceptions. Some recent survey‐based evidence suggests that consumers perceive fresh products as less safe in association with COVID‐19, as apparent in the study by Tamru, Hirvonen, and Minten (2020) for Ethiopia. In Europe and the United States, such perceptions plus awareness that better nourishment makes people less vulnerable to the virus have led to shifts in food demand from animal‐sourced toward plant‐based food products.^17^ However, the evidence is too scarce as yet to be able to make proper assumptions about such shifts in consumer preferences, and hence, they are not accounted for in the scenario analysis.

### 4.4 Policy responses

Due to their limited actual role, we did not include specific export restriction measures regarding food products (see Section 2 and the IFPRI Food Trade Policy Tracker). The present scenario does account for the substantial economic stimulus packages being implemented by most high‐income countries, including significant income transfers to households. For the OECD countries, except Mexico, Chile, Israel, and Turkey, we assume a stimulus package of, on average, 3.2% of GDP. The fiscal stimulus is introduced in the form of higher net income transfers (or lower income taxes) from the government to the representative household.

Because of the paucity of information about stimulus packages in the rest of the world, and a concern that some of what is reported may be an exaggeration of the extent of new stimulus provided, we have omitted the impacts of fiscal stimulus in the rest of the world. We are thus measuring the unmitigated impact of the shock to help calibrate policy responses, rather than an assessment of the consequences after mitigation policies have been implemented.

## 5 SCENARIO RESULTS

### 5.1 Global macroeconomic impacts

Under the given assumptions, we conclude that COVID‐19 will result in a severe global recession with global GDP falling by 5%^18^ in 2020. This COVID‐19 recession looks likely to be much deeper than that seen during the global financial crisis of 2008–2009. The economic fallout in the initial epicenters of the pandemic (China, Europe, and the United States) is also severely hurting net commodity‐exporting developing countries through declines in trade and other commodity prices, restrictions on international travel and freight, compounding the economic costs of poorer nations’ own COVID‐19‐related restrictions on movements of people and economic activity. We consider first the macroeconomic impacts and then the effects on poverty.

For developing countries as a group, we project the economic fallout to lead to a decline of aggregate GDP of 3.6% relative to 2019, but economies in Central Asia, Africa, Southeast Asia, and Latin America would be hit much harder due to their relatively high dependence on remittances, trade, and/or primary commodity exports. The recession is expected to be less severe in China and the rest of East Asia, where—with the present scenario assumptions—we expect the economic recovery to start sooner with the earlier lifting of containment measures.

We expect harsh economy‐wide impacts in sub‐Saharan Africa with GDP falling on average by almost 9% from the previous year, although agri‐food sectors may be spared and could even expand, as the collapse in export earnings and remittance incomes,^19^ with domestic production rising in light of reduced ability to import food push. Lower labor demand in urban service sectors may push workers to return to agriculture, also contributing to greater domestic food production. With more workers in the sector, however, individual incomes would remain low.

### 5.2 Poverty impacts

**FIGURE 3:**
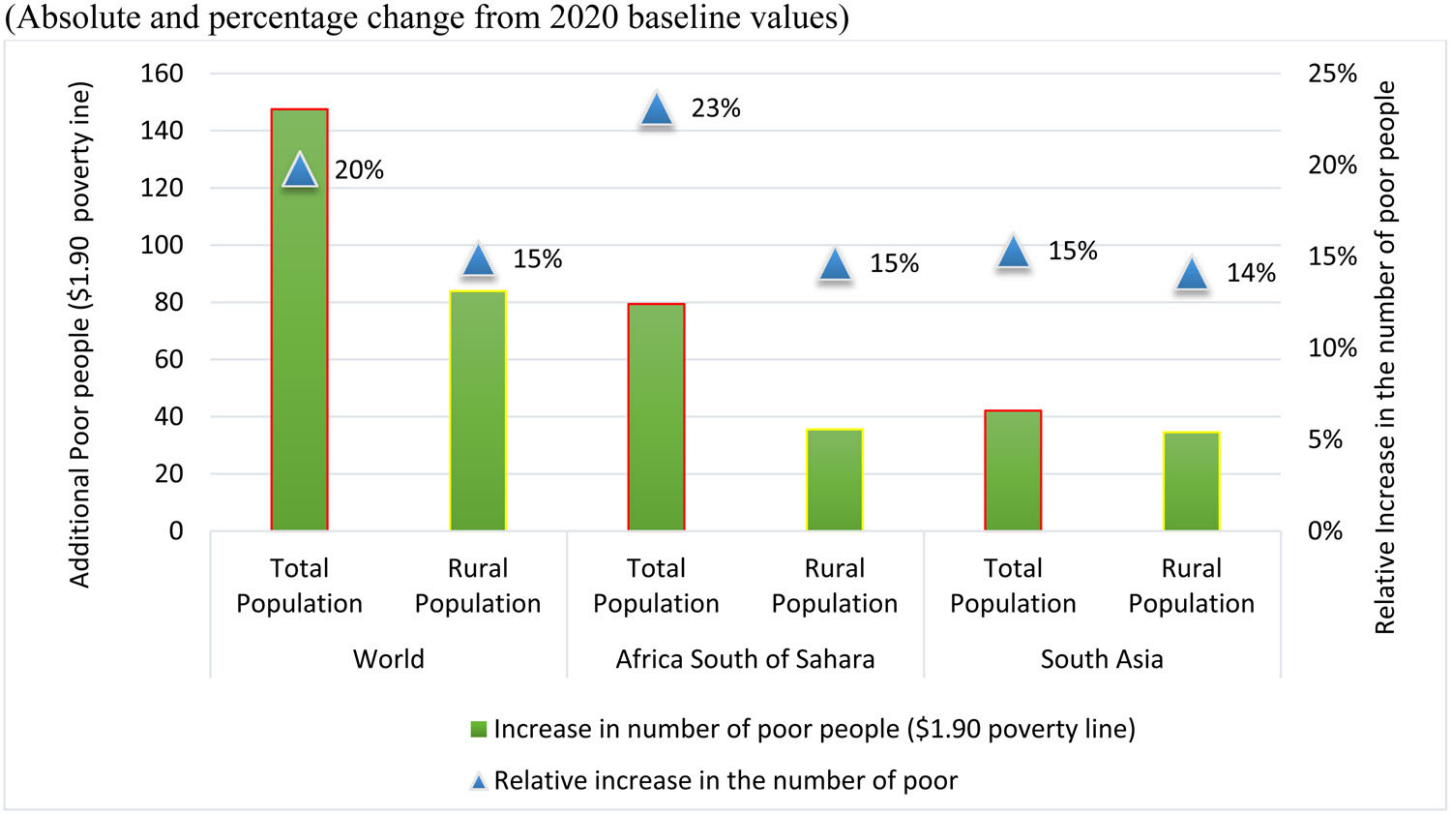
Global and Regional Poverty Impacts of MIRAGRODEP‐COVID 19 scenario (April 2020) by selected regions (Absolute and percentage change from 2020 baseline values) [Color figure can be viewed at wileyonlinelibrary.com] *Source*: MIRAGRODEP and POVANA Simulations.

Without social and economic mitigation measures such as fiscal stimulus and expansion of social safety nets in the global South (scenario assumption), the impact on extreme poverty (measured against the PPP$1.90 per person per day international poverty line) is devastating as shown in Figure 3. The number of poor increases by 20% (almost 150 million people) with respect to the situation in the absence of COVID‐19, affecting urban and rural populations in Africa south of the Sahara the most, as 80 million more people join the ranks of the poor, a 23% increase. The poverty increase in rural areas is expected to be smaller than that in urban areas, partly because of the lower rate of transmission of the disease and partly because of the robustness of demand and supply for food relative to many other, more vulnerable sectors. Accordingly, we estimate that, in sub‐Saharan Africa, the number of poor people could increase by 15% in rural areas, but as much as 44% in urban areas. In this scenario, the number of poor people in South Asia is projected to increase by 15% or 42 million people.

In both cases, the impacts on rural populations are smaller because the direct impact of COVID‐19 on agriculture is less severe than on other sectors. As these estimates refer to the numbers of extremely poor people, that is, those who typically lack the means to buy enough food, we expect a commensurate rise in the number of food‐insecure people. The ability to distinguish the reduced sensitivity of rural households to COVID‐19 is an important advantage of the more complex framework used in this study. Applying uniform income declines to the initial distribution of income will almost always result in larger poverty increases for rural people because their initial incomes are so much lower than those of urban residents in developing countries.

The estimated income declines due to COVID‐19 are much larger than seen in many earlier studies such as in Vos, Martin, and Laborde (2020), Mahler et al. (2020), and World Bank (2020a) and in most of the scenarios considered in McKibbin and Fernando (2020). However, they are substantially below the (uniform) income declines of 20% considered as an upper bound in Sumner et al. (2020). The estimates in this study fall within the range of studies surveyed in Appendix Table A.5.

### 5.3 Changes in diets and impacts on nutrition

**FIGURE 4:**
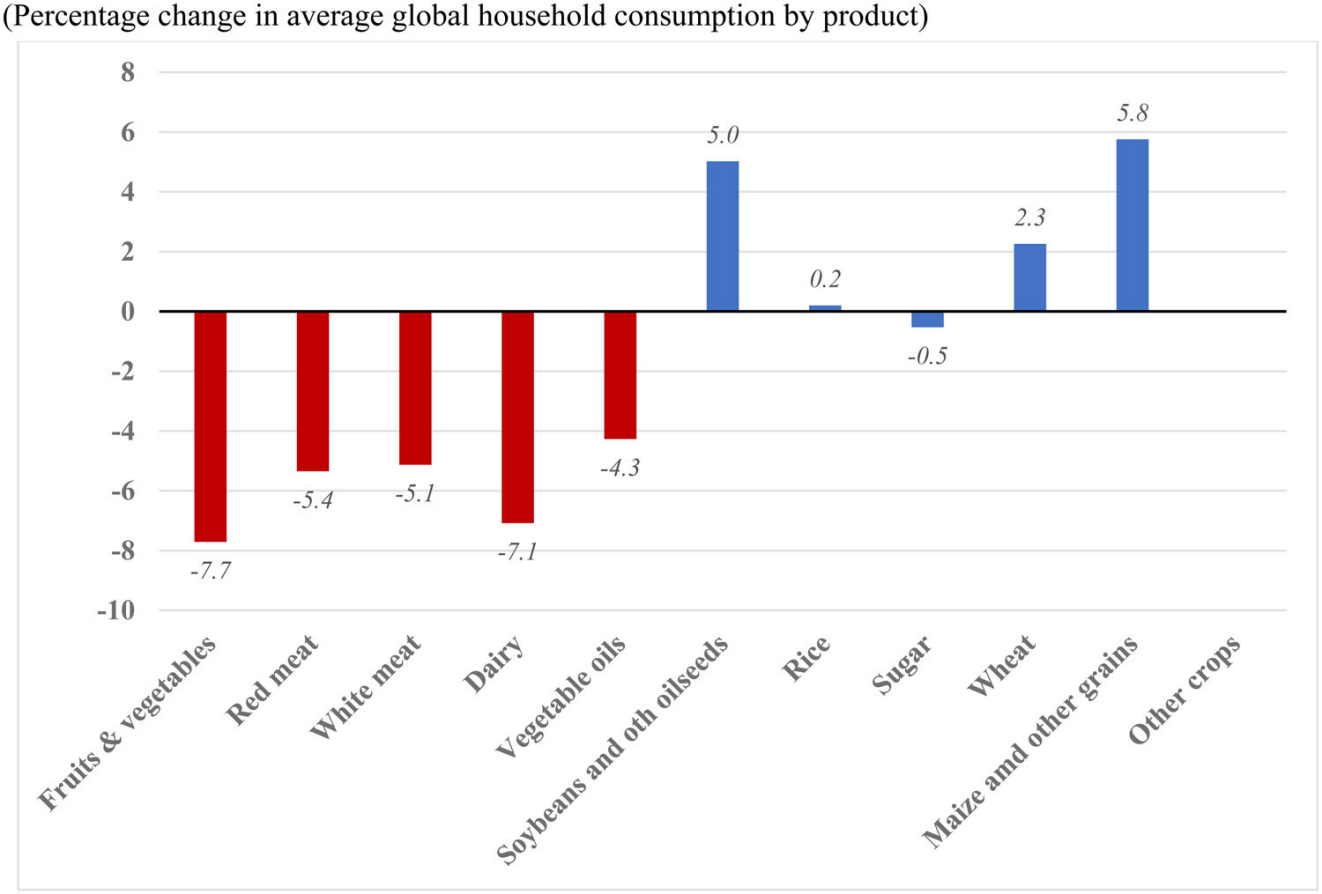
COVID‐19 impacts on diets (average effect for world) (percentage change in average global household consumption by product) [Color figure can be viewed at wileyonlinelibrary.com] *Source*: MIRAGRODEP Simulation (April 2020 scenario). *Note*: Global average based on weighted changes at the estimated at the country or regional levels. Weights are based on base value of consumption, while changes are computed on the evolution of the volume of consumption for each national representative household.

The income and price changes associated with the pandemic are likely to result in some quite substantial changes in patterns of food consumption, with adverse nutritional consequences. The declines in income and supply disruptions are likely to cause quite substantial shifts in demand away from nutrient‐dense foods such as fruits and vegetables, dairy products, and meats, and toward basic staple foods such as rice, maize, and other basic grains. Figure 4 confirms this as a global pattern. The dietary shift is (on average) similar in both developed and developing regions. The changes in consumption can be considerably sharper at the country level, as shown in Figure A.4 online.

### 5.4 Decomposition of impacts by main drivers

Given the multiple shocks used for these simulations, it is useful to understand which shocks influence the simulated outcomes the most. Not only does this provide insights into the driving forces behind both the macroeconomic and poverty outcomes, but also it allows a comparison of our approach relative to the much simpler approach of simply reducing consumption uniformly in line with the decline in GDP at constant prices used by Sumner et al. (2020), Mahler et al. (2020), and World Bank (2020b). The decomposition was done by deleting one shock at a time from the full simulation and assessing the impact of that shock. Adding up these effects provides a good estimate of the total impact and allows a decomposition of the total effect into its sources.

**FIGURE 5:**
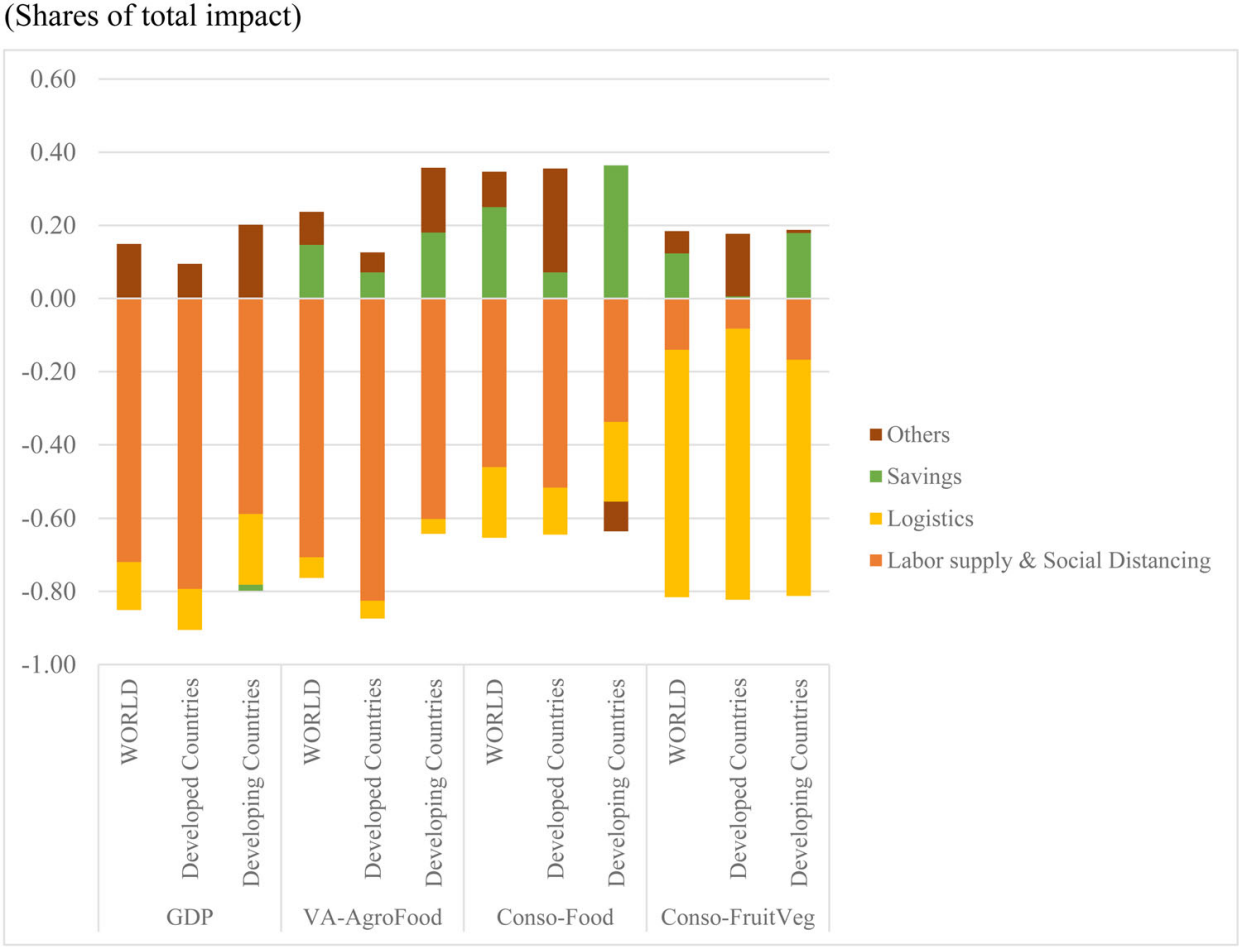
Decomposition of the simulated macroeconomic impacts by main transmission channel [Color figure can be viewed at wileyonlinelibrary.com] *Source*: MIRAGRODEP simulations results (April 2020 scenario). *Note*: Each bar in the graph represents 100% of the change in each variable in the COVID‐19 scenario and shows for each driver's positive or negative contribution (in percentage shares) to the overall change.

The first three bars in Figure 5 show that the dominant influence on the loss of aggregate GDP due to the pandemic is the reductions in labor supply, both from individual health‐related responses and from social‐distancing policies. Disruptions in logistics and the savings adjustment play small to negligible roles in the declines in GDP. The second group of bars shows the decomposition for the impacts on agri‐food sector GDP. Again, reductions in supply are primarily driven by reductions in labor availability, although these are less important than for the whole economy because a large share of agricultural value‐added is treated as essential. The savings adjustment mitigates the impact on food consumption and hence also on agri‐food production.

Income losses owing to the pandemic's direct impact on people's ability to work and that of the social distancing measures also explain most of the reduction in total food consumption, compounded by supply disruptions raising the logistical costs embedded in food prices. The savings adjustment is a mitigating factor. The increases in logistical costs affect demand for fruits and vegetables most strongly, outweighing income losses through social distancing; most notably in developing countries.

The estimates in this study fall within the range of studies surveyed in Appendix Table A.5.

Figure 5 further shows that the adjustment rule regarding private savings mitigates the macroeconomic impact of the recession on overall household consumption.^20^ The mitigating effect on consumption is generally stronger developed than in developing countries whose, on average, much poorer economic actors have less capacity to absorb the shock by drawing on own savings.

These results show that different shocks have different impacts on the different outcomes, with the direct reductions in labor having the largest impacts on GDP, whereas reductions in saving have important impacts on consumption, and increases in the cost of logistics in food supply chains having the greatest impact on consumption of fruits and vegetables.

**FIGURE 6:**
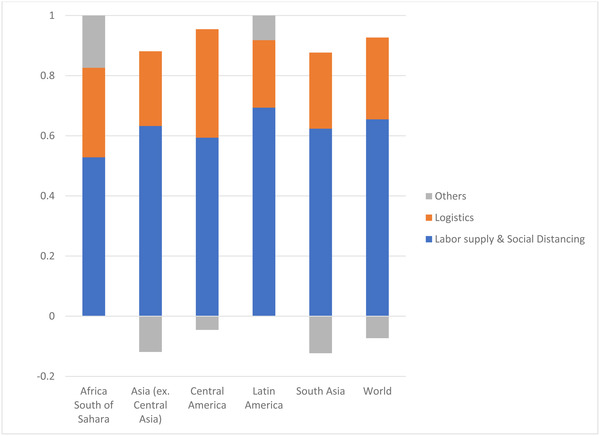
Simulated changes in extreme poverty by cause (shares of total impact) [Color figure can be viewed at wileyonlinelibrary.com] *Source*: MIRAGRODEP simulations results (April 2020 scenario). *Note*: Each bar in the graph represents 100% of the change in each variable in the COVID‐19 scenario and shows for each driver's positive or negative contribution (in percentage shares) to the overall change.

Figure 6 provides a decomposition for the total poverty impacts parallel to that for the macroeconomic impacts presented in Figure 5. Not surprisingly, it shows that the reductions in employment and in labor supply and social distancing have the largest impacts on poverty. Logistical costs have the second largest impacts, while other influences, such as oil price changes and changes in savings and investment, reduce the total increase in poverty in several regions.

**FIGURE 7:**
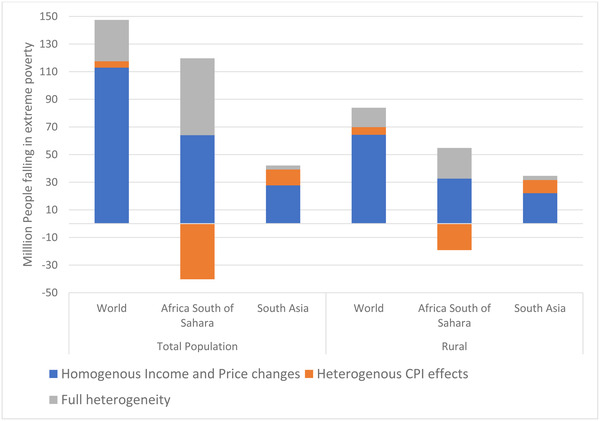
Decomposing the simulated changes in extreme poverty owing to COVID‐19 by average income and distributional shock (shares of total impact) [Color figure can be viewed at wileyonlinelibrary.com] *Source*: MIRAGRODEP simulations results (April 2020 scenario). *Note*: Each bar in the graph represents 100% of the change in each variable in the COVID‐19 scenario and shows for each driver's positive or negative contribution (in percentage shares) to the overall change.

To illustrate the difference between our approach and other studies assessing the poverty impact of the pandemic, we decompose in Figure 7 the change in the poverty rate into three components. The first, shown in the blue bar, is the impacts of average changes in incomes and in the cost of living on household real incomes. The second incorporates the nonneutral impacts of the COVID‐19 shocks on the cost of living to each household and the consequent impact on household incomes. The third considers, in addition, the nonneutral impact of the shocks on households’ individual incomes. It takes into account, for instance, the fact that many workers supplying unskilled labor—which is assumed to be the situation of the poorest—are unable to work remotely, and hence generally suffer greater income losses than higher income workers, both through the quantity of labor they can supply and the wage rates they receive.

It is clear from Figure 7 that the traditional estimate of the poverty impact of the pandemic—the observed changes in real incomes resulting from changes in average nominal incomes and consumer costs—explain most of the changes in poverty. At the global level, these uniform changes explain just over 110 million of the nearly 150 million increase in poverty. In sub‐Saharan Africa, both the uniform income effect and the differential impact on the incomes of the poor raise poverty, but this is substantially offset by many poor people being lifted out of poverty by declines in their idiosyncratic costs of living. This benefit, likely largely driven by declines in farm prices, explains why the increase in poverty observed in Figure 3 is so much smaller in Africa than in South Asia. The pattern for changes in rural poverty follows closely that observed for overall poverty.

## 6 A SCENARIO UPDATE

In previous sections, we discussed at length the analytical framework used to assess the macroeconomic and poverty impacts of the COVID‐19 crisis and described the contributions of the different drivers to the outcomes for poverty and food insecurity. That reference scenario was elaborated in April 2020, based on our observations and interpretations of the world economy, the health crisis, and the mitigation options taken up to that point in time. Although our basic methodology has not changed, new information available by the final quarter of 2020 about COVID‐19 effects on social distancing, labor supply, and policy responses differs in a number of respects from used the underpin the assumption of the original reference scenario.

To illustrate the changes in information and approach over that time, we provide an updated scenario, based on new information available for the period up to September 2020, using updated assumptions as summarized in Table 2. For health effects, we shifted from the estimates in the epidemiological model of Imperial College (Walker et al., 2020) to that of the London School of Hygiene and Tropical Medicine (Pearson et al., 2020) that provides greater detail on pandemic mitigation options adopted by countries around the world. We further rely on Google Mobility reports (Google, 2020) to track the evolution of social distancing intensity and the changes in face‐to‐face services (e.g., mobility to recreation location). Also, more recent macroeconomic assessments, such as the ADB Economic Outlook (ADB, 2020), allow us to update the assumptions about changes in consumption behavior and participation to labor markets, and the value of some specific parameters (e.g., number of workday losses) under varying mitigation strategies adopted by countries.

**TABLE 1:** Macroeconomic impacts of MIRAGRODEP‐COVID 19 scenario (April 2020) by country and country group, 2020

	(*(Percentage change from previous year)*				
	*Real household consumption*	*Real GDP*	*Agri‐food GDP*	*Exports*	*Agri‐food Exports*
**World**	−1.0	−5.1	−1.8	−17.9	−24.8
**Developed countries**	−0.2	−6.2	−3.1	−19.3	−20.3
United States and Canada	2.0	−5.8	−4.7	−21.5	−29.8
EU	−3.4	−7.6	−2.8	−40.5	−27.5
**Developing countries**	−2.5	−3.6	+0.1	−16.0	−30.5
Africa South of Sahara	−3.2	−8.9	3.9	−31.2	−20.7
North Africa & Middle East	−4.0	−6.4	1.1	−28.6	−34.6
Asia (ex. Central Asia)	−3.9	−4.6	−1.4	−23.3	−31.0
East Asia					
*China*	−4.2	−4.5	−1.7	−21.8	−29.2
South Asia	−3.7	−5.0	−2.0	−22.9	−30.7
*India*	−3.9	−5.9	−2.2	−21.8	−30.8
South‐East Asia	−4.2	−7.0	−2.8	−23.9	−31.9
Central Asia	−4.1	−9.9	2.0	−21.6	−8.3
Latin America & Caribbean	−4.4	−5.9	−3.9	−27.5	−28.5
Central America	−6.2	−8.7	−5.7	−20.2	−30.7
Rest of LAC	−4.4	−5.7	−3.9	−27.5	−28.2

*Source*: MIRAGRODEP Simulation.

*Note*: Regions in bold aggregated results computed postsimulations, weighted by the relevant country‐level variable. Details for rich countries are omitted. Real consumption is limited to household private consumption and defined as the equivalent variation (welfare).

*Note*: Regions in bold aggregated results computed postsimulations, weighted by the relevant country‐level variable. Real household consumption is measured as the “equivalent variation” of welfare. Real GDP is computed following national accounting principles. Fisher price indices between base prices and simulation prices are used. Exports of goods and services are measured FOB at constant international dollars but final export prices.

**TABLE 2:** Comparison of key assumptions for April and September 2020 MIRAGRODEP‐COVID 19 scenarios

	Scenario	
	April 2020	September 2020
Health and pandemic projections	Imperial College (Walker et al., 2020); March 26th version	London School of Hygiene and Tropical Medicine (Pearson et al., 2020); June 5th, 2020 version)
Health (nonpharmaceutical) mitigation policies	Imperial College, “Social distancing of the whole population” scenario for all countries	Countries mapped to 10 types of responses based on policy descriptions and mobility metrics (Google, 2020; per August 4th, 2020)
Social distancing parameter (e.g., number of workdays lost)	12 weeks of confinement in each country, except for Africa (8 weeks)	Adjusted allowing for country specificities within region (see above)
Value chain disruptions	Postharvest losses for perishable products: +5 points	Postharvest losses for perishable products: +5 points
Transportation and logistics	5% reduction in total factor productivity (TFP) in transport sector	5% reduction in total factor productivity (TFP) in transport sector
Preference shifter for face‐to‐face services	Uniform 25% “shadow tax” equivalent	Country‐specific “shadow tax,” scaled to social distancing intensity (tax ranging between 13% and 45%)

The changes in results for macroeconomic outcomes, agri‐food value‐added, and poverty are shown in Table 3. Although the scenarios are broadly similar in terms of the nature of the drivers, the magnitudes of the shocks have been updated and made more country‐specific. The broad upshot is that the global recession is expected to be even deeper in 2020 (a 7.1% decline in global GDP instead of a 5.1% decline). The revised assumptions do not change the earlier expectation that the agri‐food sector has held up relatively well, showing resilience compared to the rest of the economy. Globally, the agri‐food sector could even expand as agricultural production has remained relatively stable, whereas costs are down with the drop in prices for manufacturing and services.

**TABLE 3:** Poverty and macroeconomic impacts of MIRAGRODEP‐COVID 19 scenarios for 2020 (April and September 2020 scenarios)

	MIRAGRODEP COVID‐19 scenario	
	April 2020	September 2020
Real GDP (percentage change from previous year)		
World	−5.1	−7.1
Low‐ and middle‐income countries	−3.6	−5.5
Africa, South of Sahara	−8.9	−5.8
South Asia	−5.0	−12.9
Agri‐food real value‐added (percentage change from previous year)		
World	−1.8	2.5
Low‐ and middle‐income countries	0.1	2.3
Africa, South of Sahara	3.9	2.0
South Asia	−2.0	0.1
Changes in extreme poverty ($1.90 pp/pd poverty line, millions of people; changes from baseline)		
Low‐ and middle‐income countries	147.5	149.7
Africa, South of Sahara	79.4	50.5
South Asia	42.1	72.5

*Source*: MIRAGRODEP and POVANA simulations (April and September 2020 scenarios).

The aggregate findings of the updated scenario for global poverty are practically unchanged, with the number of poor expected to rise by just under 150 million. However, the regional distribution of poverty increases differs substantially from that presented in the previous sections. In the new scenario, the economic crisis is expected to be deeper than previously anticipated in South Asia, particularly in India, and milder in Africa. As a result, this simulation projects a smaller, though still significant increase in poverty sub‐Saharan Africa (50 million instead of near 80 million) and the larger increase affecting people in South Asia (72 million instead of 42 million).

## 7 CONCLUSIONS

The key goal of this paper was to provide a rigorous framework to assess the risks pandemics like COVID‐19 pose to global poverty and food security. Accordingly, we first considered the nature of the relationships between the COVID‐19 pandemic and the overall economy. This made clear that the major impacts of the pandemic on poverty and food security are more likely to come from shocks to household incomes, and hence to food access, than from impacts on food markets directly. However, we recognize that there are important direct impacts of the disease on food markets, particularly in the more labor‐intensive parts of the food chain, and in areas such as food services, where the need for social distancing is sharply reducing the operation of restaurants.

Given the multiplicity of links between the pandemic, household incomes, and food security, we concluded that a framework linking economy‐wide modeling with household models was needed to capture the impacts of the shock on poverty. We used the MIRAGRODEP global CGE model linked to epidemiological models to capture the impacts on the global economy, and the POVANA household models to capture the impacts at the household level.

The simulation experiments were designed to capture the impacts of the crisis begin with the direct, thus far seemingly minor, impacts of the disease on labor supply resulting from increases in morbidity and mortality. The next key shock was the impacts of social distancing, whether undertaken out of concern about catching the disease or as part of a concerted policy of suppressing the disease—a very important channel of effect with highly specific impacts by sector and type of labor. In addition, we considered the impacts of increases in logistical costs associated with the disease.

Our initial results suggested that COVID‐19 would cause a decline in global GDP of about 5% in 2020, with a similar decline in South Asia and a larger decline (−9%) in Africa South of the Sahara, and much larger declines in global trade because of both increases in logistical costs and declines in investment as consumers and governments seek to reduce the adverse impacts of the crisis on living standards by reducing private and government savings. Consumers are also expected to have shifted their food purchases, buying less nutrient dense, but more expensive, products such as fruits and vegetables, meat, and dairy products, and buying more calorie‐rich and cheaper cereals and processed foods. In an updated scenario, however, using new information about—*inter alia*—the spread of COVID‐19 and related social distancing measures, particularly taking into account the reduced estimates of the spread of the disease in Africa, we expect that the global recession could be steeper than previously anticipated, driven in part by a much stronger economic decline in South Asia.

To better understand these results, we decomposed them by major drivers. The economic consequences of reduced labor supply and social distancing drive most of the impacts on GDP worldwide. Fiscal stimulus in high‐income countries and declines in private savings mitigate some, but far from all, the adverse impact on total and food consumption.

The analysis concludes that the pandemic will likely increase the number of people in poverty by about 150 million people, or 20% of current poverty levels. In our reference scenario, most of this increase in extreme poverty was expected to occur in Africa South of the Sahara and South Asia, where many people are currently close to this poverty line. An updated analysis suggests that the increase in poverty may be smaller than originally anticipated in Africa and larger in South Asia, with the global total impact remaining very similar at just under 150 million.

The analytical framework that we use captures many important nonneutralities in the effects of the crisis that are ignored in simpler analyses assuming that all incomes change equally. For example, we find that poverty increases are likely to be smaller, both in absolute numbers and relative to current poverty rates, in rural areas that are likely less hard hit by the crisis. An analysis of these poverty results suggests that accounting for just the average changes in incomes and in consumer prices would capture only about three‐quarters of the total impact of the crisis on poverty rates. Many of the impacts are nonneutral between the poor and the rich and outcomes for the poor are, on average, substantially worse for higher income and more educated people, many of whom can continue to work productively at a distance.

The actual implications of COVID‐19 for poverty and food security will depend on a wide range factors, many of which are simply unknown at this point—such as resurgence of the disease during the northern winter and spring, and the efficacy and adoption of potential vaccines. Thus, the results in this paper should not be taken in any way as a precise forecast of the outcome. Rather, the paper provides an approach for evidence‐based “what‐if” scenario analysis of the impacts of broad‐based shocks such as COVID‐19 for poverty, food insecurity, and dietary change. As such it should help better understand the relative importance of the multiple channels of transmission and inform policymakers about the socioeconomic consequences of mitigation measures taken to reduce public health risks, and hence, the potential trade‐offs between efforts to safeguard lives and those to protect livelihoods.

## Supporting information

Figure A4 COVID‐19 impacts on diets in China and Nigeria
Table A2: Coverage of household surveys in POVANA database
Table A.5. Estimated Impacts of COVID‐19 on GDP and on Poverty

## Footnotes

1
http://iresearch.worldbank.org/PovcalNet/.

2 See https://www.imf.org/en/Topics/imf‐and‐covid19/Policy‐Responses‐to‐COVID‐19#I

3 See the World Bank's “living paper” at https://tinyurl.com/yd4g4z45

4 Up‐to‐date counts are available at IFPRI's food trade policy tracker.

5 Modelling International Relations under Applied General Equilibrium model enhanced for the AGRODEP modeling consortium (http://www.agrodep.org/models/library).

6 See Online Appendix A.2 for the coverage of the household survey data used for the present analysis.

7
https://www.ifpri.org/spotlight/ifpri‐resources‐and‐analyses‐covid‐19‐also‐known‐coronavirus

8 In the updated scenario (discussed in Section 6), we use alternative projections for the spread of COVID‐19 of the epidemiological model of the London School of Hygiene and Tropical Medicine (LSHTM) (Pearson et al., 2020).

9 See Online Appendix A.3 for the procedure for deriving this estimate.

10 American Transportation Research Institute; see, for instance, https://tinyurl.com/yxkr92g6

11 Reuters, ‘West African food trade under strain as COVID‐19 shuts borders’, May 27, 2020. https://tinyurl.com/yxzjt9fm

12 We use a shadow tax instead of a preference shifter in the model to avoid changing the utility function that would compromise the welfare analysis.

13 For comparison, oil prices for WTI Crude contracts declined by 33% between June 2019 and June 2020 (from US$53 to US$35 per barrel) and by 35% between the start of 2020 and November 10 of the same year (from US$62 to US$40), after showing a steep decline between January and the end of April and a slow recovery. Since the model combines natural gas and crude oil into one variable, the simulated decline in global energy prices is somewhat lower than the observed 35%, given that the impact of the supply and demand shifts on the price of natural gas has been smaller than that on oil.

14 World Bank, World Development Indicators, https://databank.worldbank.org/reports.aspx?source=world‐development‐indicators#, accessed 24 July 2020.

15
https://fred.stlouisfed.org/series/PSAVERT

16
https://fred.stlouisfed.org/series/B057RC1Q027SBEA

17
https://tinyurl.com/y4w97xee

18 This decline is relative to 2019 levels. Relative to the 2020 baseline (counterfactual without the COVID‐19 shock), this implies a 7% decline in global GDP. Only in Table 1, do we present the macroeconomic impacts relative to the previous year (for ease of comparison with other estimates and projections). All other simulation results are with respect to the 2020 baseline (counterfactual without the COVID shock).

19 Remittance incomes make up more than 10% of gross foreign exchange earnings in sub‐Saharan Africa. In the model, we assume the region's earnings from remittances drop by 8%. Recent projections project a decline of 9% for 2020 (World Bank 2020c).

20 It is important to point out that the impact on consumption is softened further in the model estimations because GDP is measured in real terms through a Fisher index, while the impact on consumption is measured through a welfare metric (equivalent variation) typically used in CGE models.
