# Supplementary material for: Impacts of COVID‐19 on global poverty, food security, and diets: Insights from global model scenario analysis
Source: Agric Econ. 2021 Apr 8;52(3):375–90. doi: 10.1111/agec.12624 (PMC8251321; doi:10.1111/agec.12624)
Supplement: Supplementary file 1 — Figure A4 COVID‐19 impacts on diets in China and Nigeria Table A2: Coverage of household surveys in POVANA database Table A.5. Estimated Impacts of COVID‐19 on GDP and on Poverty [file AGEC-52-375-s001.docx]

***Supplementary Material to***

**Impacts of COVID-19 on global poverty, food security and diets: Insights from global model scenario analysis**

**David Laborde^1^, Will Martin^1^, and Rob Vos^1^**

*^1^ International Food Policy Research Institute, Washington D.C., USA*

**Correspondence:**

Will Martin, International Food policy Research Institute, 1201 I Street N.W,, Washington D.C. 20005, USA, Email: [w.martin@cgiar.org](mailto:w.martin@cgiar.org)

(*Final revisions following acceptance, 4 December 202*0)

**Online Appendices A.1–A.5**

## **Appendix A.1: The Integrated Modeling Framework: MIRAGRODEP and POVANA**

## **A.1.1 The MIRAGRODEP model**

MIRAGRODEP is a global Computable General Equilibrium (CGE) model based on MIRAGE (Decreux & Valin, 2007). The model was developed and improved with the support of the African Growth and Development Policy Modeling Consortium (AGRODEP). It is a multi-region, multi-sector, dynamically recursive CGE model. The model allows for a detailed and consistent representation of the economic and trade relations between countries (Laborde, Robichaud & Tokgoz, 2013). In each country, a representative consumer maximizes a CES-LES (Constant Elasticity of Substitution-Linear Expenditure System) utility function subject to an endogenous budget constraint to generate the allocation of expenditures across goods. This functional form replaces the Cobb-Douglas structure of the Stone-Geary function (that is, LES) with a CES structure that retains the ability of the LES system to incorporate different income elasticities of demand (Stone, 1954), with those for food typically lower than those for manufactured goods and services, while attenuating the strong link between income and price elasticities characteristic of the LES. The demand system is calibrated on the income and price elasticities estimated by Muhammad et al. (2017). Once total consumption of each good has been determined, the origin of the goods consumed is determined by another CES nested structure, following the Armington assumption of imperfect substitutability between imported and domestic products.

On the production side, demands for intermediate goods are determined through a Leontief production function that specifies intermediate input demands in fixed proportions to output. Total value added is determined through a CES function of unskilled labor and a composite factor of skilled labor and capital. This specification assumes a lower degree of substitutability between the last two production factors. In agriculture and mining, production also depends on land and natural resources. In the present application of the model, we assume that new capital investment is perfectly mobile across sectors, while installed capital is immobile. Furthermore, skilled labor is assumed to be fully mobile across sectors, while unskilled labor is only partially mobile between agricultural and non-agricultural sectors. Due to the, presumed, short-term nature of the COVID-19 shock, we divide the original substitution elasticity for factors of production in the production tree by a factor of two, as substitution effects tend to be smaller in the short run. Indeed, we allow producers very little ability to change the capital-labor utilization ratio within a single year.

For the present scenario analysis, we assume further that investment is savings-driven in each country and, hence, will fall with any drop in savings. The real exchange rate is assumed to be flexible, that is, it adjusts endogenously such that the current account balance of the balance of payments remains constant as a share of each individual country’s GDP. It implies we also assume that foreign savings are fixed as a share of GDP. To guarantee the supply of external finance matches demand in the global capital market, capital inflows towards countries with a current account deficit are “scaled” up or down by a homogenous factor to capture the scarcity or abundance of international capital. Hence, we assume portfolio preferences and capacity to borrow on international markets remain constant for all countries.

For the present analysis, we do not consider endogenous tax policy responses by governments. Instead, we consider that, except for those countries where we model a budgetary policy response (such as the economic stimulus measures taken by many of the richer nations; see the section on scenario assumptions in the paper), a reduction in tax receipts is associated with a reduction in public spending, thus keeping the public deficit/surplus to GDP constant. This default assumption is used to avoid creating apparent welfare gains by supporting current consumption through an increase in public debt without considering the future welfare costs of the debt.

As in Laborde and Martin (2018), we use the GTAP 9.1 database as MIRAGRODEP’s main source of data and parameters. This database allows us to readily use up to 140 regions/countries and 65 products and production sectors. In addition, the database is enhanced by datasets on land use, agricultural production, food balance sheets, agricultural domestic support measures and trade policies, as well as updated Social Accounting Matrices for all individually specified countries. A realistic baseline is constructed aligned with the United Nations’ demographic projections and updated IMF economic growth estimates and projections to bring the base year values (2011) to those of the actual year of simulation (2020) and the preceding year (2019).

For this specific study, we condense the model to 29 sectors, of which 18 are related to agri-food activities (primary production and downstream activities) and 36 regions/countries.^[[1]](#footnote-1)^ For a given year (2020 in the present case), the model consists of 310,345 equations and (non-zero) variables.

## **A.1.2 The POVANA framework**

To translate the CGE model simulation results into poverty impacts, we rely on the POVANA dataset and follow an approach like Ivanic and Martin (2018). The coverage of the POVANA dataset and the most recent documentation is available online^^[[2]](#footnote-2)^^ and the survey coverage for the present analysis is specified in Online Appendix A.1.3 below. For the sake of comparison using the most recent peer-review publications on the topic, we use the same version of the POVANA dataset as in Laborde and Martin (2018).

While the household coverage, largely composed of LSMS survey data, for 31 countries, includes more than 300,000 representative households, we retain and use directly the information available from household surveys on the income sources and expenditure patterns in each of just over 285,000 sample households. Our approach requires consistency between the expenditure and income information for each household, and we adjust where possible to reconcile data across sources. However, household records requiring very large adjustments for this reason were excluded.

Online Appendix A.1.3 below explains more formally how the global CGE findings are linked to the POVANA household model. Intuitively, there are two key linkages between the macro findings and the household models. The first is through exogenous shocks, such as changes in farm productivity, that are imposed at both the economy-wide and the household level. The second is through changes in prices and wages that are endogenous to the economy-wide model and imposed on the household model. A simple example of a direct effect is a decline in labor supply due to illness that exogenously lowers the household’s labor supply. Another relatively simple case arises from a shock that results in a sizeable change in the price of a food commodity for which a poor household is a net seller or net buyer. The short-run effect of such a shock on real household incomes can be estimated with information in the POVANA database on the degree to which households are net sellers or net buyers of the product.

## Appendix A.1.3: Linking MIRAGRODEP to the POVANA model

To understand the poverty estimates reported in this study, we first determine the nominal income (*x*) of household *i* as

$x_{i}=\pi_{i}\left( \vec{p},\vec{p}^{*} \right)+\vec{w}_{i}´\vec{v}_{i}+t_{i}+\phi_{i}+\rho_{i}$

where $\pi_{i}$ is the profit function for any unincorporated business activities of household *i*, defined over a vector of output prices $\vec{p}$, input prices (goods and wages) $\vec{p}^{*}$. Output quantities $\vec{q}_{i}$ and input demands (hired labor and other inputs in terms of good and services) $\vec{r}_{i}$ are implicit in this profit function, and $\vec{q}_{i}$ is related to input quantities through the production relation $\tau_{i}$ such that $\vec{q}_{i}=\tau_{i}(\vec{r}_{i})$. Household income may also be derived from labor and other factors supplied, with this income represented by the inner product of $\vec{w}_{i}$and the vector of factors sold by the household $\vec{v}_{i}$. Other sources of household income include net public transfers received/paid by the household $t_{i}$; the net international remittances received/paid by the household $\phi_{i}$; and other net domestic private transfers $\rho_{i}$. Similarly, expenditures including self-consumption, are defined by the expenditure function $e_{i}\left( u_{i},\vec{p}^{*} \right)=\vec{c}_{i}\vec{p}^{*}$with $\vec{c}_{i}$ the vector of quantities consumed.

Initially, we check that $x_{i}=e_{i}+s_{i}$, where $s_{i}$ represents the savings of household i. In this simulation, we consider that $s_{i}$ at the household level is exogenous. Indeed, we want to compute the compensation measures of welfare changes at fixed initial utility ($u_{i}$) and net savings, to avoid associating decreasing savings—a normal coping strategy—with a positive utility outcome (increased consumption). When using the extreme poverty line, households at this income level or around it, have extremely scarce available savings so this assumption is not critical in our assessment. For many households, $\rho_{i}$ (transfers) need to be adjusted to balance income and expenditures in the baseline. Please note that, as explained above, first round impacts on savings are considered in the CGE model.

Because we assume in this scenario analysis that the COVID-19 shock is short lived (the shock and the response take place within the same year), we follow Deaton (1989) and consider only the first-order impacts on $\pi_{i}$ and on $e_{i}\left( u_{i},\vec{p}^{*} \right)$. We measure changes in welfare or real income as${\Delta y}_{i}={\Delta x}_{i}+{\Delta e}_{i}+\Delta s_{i}$ to obtain the hypothetical transfer from the rest of the world needed to hold utility at its initial level following changes in nominal income arising from price or productivity changes and associated changes in the cost of living. Using hat notation, with $\hat{y}=\frac{dy}{y}$ for the proportional change in a variable, dropping the household *i* index and introducing *k* as an index over goods and services, we obtain:

$${\Delta y}=\sum_{k} \hat{p}_{k}\bar{p}_{k}\bar{q}_{k}+\sum_{k} \hat{q}_{k}\bar{q}_{k}p_{k}-\sum_{k} \hat{r}_{k}r_{k}\bar{p}_{k}^{*}-\sum_{k} \hat{p}_{k}^{*}\bar{p}_{k}^{*}\bar{r}_{k}+\hat{wu}.\bar{wu}.\bar{ku}+\hat{wm}.\bar{wm}.\bar{km}++\hat{ku}.\bar{ku}.\bar{wu}+\hat{km}.\bar{km}.\bar{wm}+\hat{t}\bar{t}+\hat{r}\bar{r}+\hat{\rho}\bar{\rho}+\sum_{k} \hat{c}_{k}\bar{c}_{k}\bar{p}_{k}^{*}+\sum_{k} \hat{p}_{k}^{*}\bar{p}_{k}^{*}\bar{c}_{k}+\hat{s}\bar{s}$$

where $vu r$epresents the labor endowment of household i sold on the labor market outside the household’s business activity at wage rate $wu$, and $vm$ is a composite of other factors of production (capital, land, etc.…) owned by the household and rented out on markets at rental rate $wm$. This general case incorporates a wide range of household circumstances. Importantly, for many poor households, the initial values of many of these variables, such as property taxes, are generally zero.

Due to the presumed short-term nature of the shock and limited coping capacities of household, we neglect a number of possible adjustment strategies, such as sales of non-labor assets ($\hat{vm}=0$), production inputs ($\hat{r}_{k}=0)$ and consumption pattern ($\hat{c}_{k}=0$). This approach allows us to obtain a first-order estimate of the welfare impact of the shock on households—providing a compensation-based money measure of the impact. It also avoids having to make specific assumptions about which coping strategy household choose to mitigate reductions in their consumption bundle. As indicated above, we further assume households do not reduce savings as a coping strategy ($\hat{s}=0$). In addition, we further assume governments do not adjust income tax rates as a COVID-19 response ($\hat{t}$=0).

Adjustments of other variables in the above specifications are endogenous as determined by the MIRAGRODEP-structural model equations. Household, firm and government behavior all vary by country (or region). For the geographical entity represented in the model to which the individual household belongs:

- $\hat{p}_{k}$ is the relative producer price change as defined in the CGE for the good or service *k*, or group of goods or services in which it is included.
- $\hat{p}_{k}^{*}$ is the relative consumer price change as defined in the CGE for the commodity *k*, or group of commodities in which it is included. This price includes the import price index based on the Armington assumption (true price index of the associated good or services at the consumer level).
- For any goods or services *y*, for which a specific household *i,* produces a significant amount for self-consumption, $\hat{p}_{k}^{*}$ is assumed to be equal to the producer price change, instead of the consumer price change.
- Due to our focus on the left tail of the income distribution, we consider that all labor is unskilled labor and $\hat{wu}$ is the relative change in the wage of unskilled workers. Since MIRAGRODEP considers two labor markets, rural and urban, $\hat{wu}$ is specified separately for rural and urban workers, depending on the household location. The location of workers remains constant in the simulation.
- Variable $\hat{wm}$ represents the relative change of payments to non-labor endowments in the CGE (country level weights), including land, capital and natural resources.
- For each household, we implement a reduction in labor supplied to the market, $\hat{ku}$, identical to the reduction of unskilled labor supply introduced in the CGE (exogenous scenario shifter) as a consequence of lockdown and/or disease. We assume that “unsold” labor by the household, captured by $\hat{wu}$, is not recycled in the incorporated business activity, leading to additional production (or, put differently: $\frac{dq_{k}}{dwu}=0$). Similarly, the initial amount of labor used internally by the household is not assumed to change due to confinement measures.
- $\hat{\rho}$ is assumed to be equal to $\hat{wm}$. Various assumptions have been experimented in the past and there is no perfect solution. The $\rho_{i}$ term captures many elements, including statistical adjustment. Leaving this constant in nominal terms would lead to a significant amount of “dark matter” in the system, stabilizing the system without any justification. While the transfers and “rents” captured by this measure may be stabilizing, assuming $\hat{\rho}=0$ would be an excessive assumption, and an inconsistent one since from a CGE point of view, no “values” should remain fixed. Any value/price should be indexed on at least one price in the system to avoid violation of Walras Law. Accordingly, we link $\hat{\rho}$ and $\hat{wm};$
- We consider that $\hat{q}_{k}$ is only impacted by the changes in labor productivity driven by the CGE model. Indeed, one of our strong assumption is that we do not consider disruption, including for hired labor, in the availability of inputs used by the household, so we do not consider changes in $\vec{r}$. Logically, no change in $\vec{r}$ does not lead to changes in $\vec{q}$. Actually, this assumption has very limited implication for the assessment of the impacts of COVID-19 on global poverty, since households living in extreme poverty which are self-employed and own a microenterprise or small farm typically rely on few assets, intermediate inputs, or hired labor, and mostly rely on the labor, administrative and management skills of their families. Still, we want to capture a productivity effect by indexing $\hat{q}_{k}$ to the relative change in output per unit of labor and by sector, to guarantee the consistency of the framework regarding relative wage changes, productivity changes and prices changes. Since, we use wage changes from the CGE that capture the evolution of the marginal labor productivity in value, due to price and productivity effect, we need to have the various elements in the framework to avoid a systematic bias about self-employment.

With these assumptions, the model estimates a new income level for each household and hence a new income distribution for each new scenario. The per capita incomes for each household in each household survey are subsequently compared with the international (extreme) poverty line of $1.90 per person, per day at 2011 purchasing power parity (PPP) dollar values (using PPP conversion factors as available at [PovcalNet](http://iresearch.worldbank.org/PovcalNet/povOnDemand.aspx) to convert the poverty line into domestic prices). The poverty rate is calculated as the share of the population with an income below the indicated poverty line. This calibration process allows us to define the nominal per capita income, ${Pline}_{r}$, at base prices associated with our poverty definition. We can also define this value as a real income poverty line at base prices. For any level of income, the number of poor people is defined by $NP=\sum_{0}^{N} w_{i}\times\delta^{i}\left( x_{i} \right)$ where  *i* is the household index, *N* the overall household set in the household survey for the country *r* , $w_{i}$ the demographic weight of this household, and $\delta^{i}\left( \right)$ a dummy variable defined on the income level indicating if the household is above or below the poverty line such as: $\delta^{i}\left( x_{i} \right)=\left\{ \begin{aligned} 0 if x_{i}>{Pline}_{r} \\ 1 if x_{i}\leq{Pline}_{r} \end{aligned} \right.$. The poverty incidence in the total population is equal to $P^{0}=\frac{NP}{\sum_{0}^{N} w_{i}}$ . We compute poverty headcount and poverty incidence for sub-groups of population, e.g. urban/rural, farmer/non farmer for instance $NP= {NP}^{Rural}$+${NP}^{Urban}$ , by changing the composition of N. But the poverty line is not specific to any group. When doing simulations, we look at the real income change of households and see how many households are actually crossing this poverty line in one direction or another, considering a new vector $\delta^{i}\left( \bar{x}_{i}+\Delta y_{i} \right)$, with ${\Delta y}_{i}$ as defined previously and $\bar{x}_{i}$ the initial income.

Our sample of countries has wide geographic coverage and includes 65% of the world’s extreme poor. In order to obtain estimates of poverty changes at the global level, we need to associate each country, not included in the POVANA sample, with a weighted vector of in-sample countries. As specified below, these weights are estimated by minimizing the quadratic distance between a vector, for the real country, defined by a set of ex-ante variables (initial level of poverty, share of rural population from the World Development Indicators) and ex-post variables (impact on GDP and farm value added), and the same vector for the weighted constructs. Combining ex-ante and ex-post elements is important since two countries with similar structural features at the macroeconomic level (poverty rate, GDP per capita, share of rural population) could be impacted differently due to various sectoral specialization, or idiosyncratic shocks (infection rates) or policy responses (confinement).

To summarize, for each country included in the POVANA dataset, we define the changes in the number of poor people as $\Delta NP=\sum_{0}^{N} w_{i}\times\delta^{i}\left( \bar{x}_{i}+\Delta y_{i} \right)-\sum_{0}^{N} w_{i}\times\delta^{i}\left( \bar{x}_{i} \right)$ . Since, the current size of the population is not significantly modified by the nature of the shock (low mortality), the changes in poverty rates are driven by the changes in the numerator, i.e. the number of poor people.

For a country *r* not included in the POVANA sample, we compute the ${\Delta NP}_{r}$ such as:

${\Delta NP}_{r}=\bar{NP}_{r}^{urban}\times\sum_{j} w_{r,j}\frac{{\Delta NP}_{j}^{rural}}{\bar{NP}_{j}^{rural}}+\bar{NP}_{r}^{rural}\times\sum_{j} w_{r,j}\frac{{\Delta NP}_{j}^{urban}}{\bar{NP}_{j}^{urban}}$ with $w_{r,j}$ is the weight of country *j* in the linear combination used for country *r,* and $\bar{NP}_{j}$the initial number of poor people in the base data for each relevant country group/countries. This approach allows variations in impacts between urban and rural people to be captured in a consistent manner.

The weighting procedure for countries not included in the household database is as follows. The distance that minimizes a weighted squared difference between country’s i specific variables and those of a group of reference countries is defined as:

$${Distance}_{i,s}=0.5*\frac{\left( \hat{{AgProduction}_{i,s}}-\sum_{j\in J} w_{i,j,s}\hat{AgProduction}_{j,s} \right)^{2}}{\sum_{k\in K} abs(\hat{AgProduction}_{k,s})/card(K)}$$

$$+\sum_{f\in F} 0.1*\frac{\left( {component}_{f,i}-\sum_{j\in J} w_{i,j,s}{component}_{f,j} \right)^{2}}{\sum_{k\in K} \frac{{component}_{f,k}}{card\left( K \right)}} \forall i\in I,\forall s\in S$$

s.t. $w_{i,j,s}\geq0$ , $\sum_{j\in J}^{w_{i,j,s}>0} 1\leq5$, $\sum_{j\in J} w_{i,j,s}=1$ and $\forall i\in I$,$\forall s\in S$

where *I* is the set of every country in the world in our global household dataset; *K* is the same set as *I*; *J* is the set of 31 countries included in our global household dataset^[[3]](#footnote-3)^ and *j* an element of *J;* $w_{i,j,s}$ is the weight of country *j* in the linear combination used for country *i;* $\hat{{AgProduction}_{i,s}}$is the relative change in the total value of agricultural production of country *i* in a given scenario *s*. *S* being the scenario space. This change is computed by combining FAOSTAT data on individual crop production and prices for each country and the quantity and price changes obtained in the CGE, either for the country, if singled out in the model, or the region to which the country belongs. This top-down approach allows us to capture, at the country level, for each country of the world, one of the key drivers, i.e. farm income, of the results for a given scenario. Indeed, we need to rely on this approach, capturing ex-post elements, since the price changes driven by the productivity changes are region, production, and scenario specific and focusing on ex-ante clustering analysis would miss this point.

${Component}_{f,i}$ stands for a set of country-level variables that are used to bring together similar countries. The set *F* includes the following variables for 2013, extracted from the World Development Indicators database: GDP per capita in PPP$ (2011), poverty incidence (measured against the PPP$1.90 pp/pd poverty line), prevalence of undernourishment, share of agriculture in total GDP, and share of rural population.

Online Appendix A.2: Household surveys used in this study

The POVANA household model uses data on the full income distribution for around 300,000 households in 31 countries. The country and survey coverages for the analysis in the present study are listed in Table A2 below. For the latest coverage of the POVANA data base, see: <https://public.tableau.com/profile/laborde6680#!/vizhome/POVANA_Surveys/POVANA>.

Table A2: Coverage of household surveys in POVANA database

| **Country name** | **Year** | **Survey name** |
| --- | --- | --- |
| Albania | 2005 | Living Standards Measurement Survey |
| Armenia | 2004 | Integrated Survey of Living Standards |
| Bangladesh | 2005 | Household Income-Expenditure Survey |
| Belize | 2009 | Household Income and Expenditure Survey |
| Cambodia | 2003 | Household Socio-economic Survey |
| China | 2002 | Chinese Household Income Project |
| Côte d'Ivoire | 2002 | Enquete Niveau de Vie des Ménages |
| Ecuador | 2006 | Encuesta Condiciones de vida |
| Guatemala | 2006 | Encuesta Nacional de Condiciones de Vida |
| India | 2005 | India Human Development Survey (IHDS) |
| Indonesia | 2007 | Indonesia Family Life Survey |
| Malawi | 2004 | Second Integrated Household Survey |
| Moldova | 2009 | Cercetarea Bugetelor de Familie |
| Mongolia | 2002 | Household Income and Expenditure Survey |
| Nepal | 2002 | Nepal Living Standards Survey II |
| Nicaragua | 2005 | Encuesta Nacional de Hogares sore Medicion de Nivel de Vida |
| Niger | 2007 | Enquete National sur Le Budget et la Consommation des Menages |
| Nigeria | 2003 | Nigeria Living Standards Survey |
| Pakistan | 2005 | Pakistan Social and Living Standards Measurement Survey |
| Panama | 2003 | Encuesta de Niveles de Vida |
| Peru | 2007 | Encuesta Nacional de Hogares |
| Rwanda | 2005 | Integrated Household Living Conditions Survey |
| Sierra Leone | 2011 | Sierra Leone Integrated Household Survey |
| Sri Lanka | 2007 | Household Income and Expenditure Survey |
| Tajikistan | 2007 | Living Standards Measurement Survey |
| Tanzania | 2008 | National Panel Survey |
| Timor-Leste | 2007 | Poverty Assessment Project |
| Uganda | 2005 | Socio-Economic Survey |
| Viet Nam | 2010 | Household Living Standard Survey |
| Yemen | 2006 | Household Budget Survey |
| Zambia | 2010 | Living Conditions Monitoring Survey |

Source: MIRAGRODEP and POVANA model database.

Online Appendix A.3: Use of epidemiological models

As explained in the main text, we consider two broad impacts on labor markets. The first is the direct impact of mortality and morbidity on labor supply. The second is the impacts on labor supply of social distancing actions needed to reduce transmission of the disease. The first impact is linked to the direct impact of the disease.

For the reference scenario, we use the estimates provided by the Imperial College for each country in the world on March 26, 2020 (Walker et al., 2020). Specifically, we use the “Social distancing of the whole population” scenario for all countries. Since their online materials do not provide results by age cohorts, we re-estimate those by considering that for a given country *r*:
 ${Number of Infections}_{r}=\sum_{c} \gamma_{r}\varphi_{c}{Pop}_{c,r}$ and ${Number of Deaths}_{r}= \sum_{c} \delta_{r}\mu_{c}\varphi_{c}{Pop}_{c,r}$ ,

where ${Pop}_{c,r}$ refers to the population size in the age cohort *c* in country *r;* $\varphi_{c}$ is the default probability of infection by age cohort; and $\mu_{c}$ is the mortality rate by age cohort. The latter two parameters are taken from observed values in existing studies,^[[4]](#footnote-4)^ while $\delta_{r}$ and $\gamma_{r}$ are calibrated for each country. This allows us to recompute consistent distributions of infections and deaths by age cohort.

To calculate the implications for the workforce, we then consider the number of cases in the active population (defined over the 15 to 65 years old population) to impact the share of working days lost days. We consider that the death of an individual occurring in month *x* of the year results in a loss of $\frac{(12-x)}{12}$ of annual labor supply; while sickness is associated with 15 days of lost labor supply (15/365). In this scenario, we do not differentiate cases by degree of severity with differentiated coefficients for infected/hospitalized/intensive-care treatments cases. The direct relative reduction in labor supply due to the disease directly is estimated as

$\hat{l_{r}}=\frac{\sum_{c\subset[15;65]} \gamma_{r}\varphi_{c}{Pop}_{c,r}\times\frac{15}{365} + \sum_{c} \delta_{r}\mu_{c}\varphi_{c}{Pop}_{c,r}\times\frac{(12-x)}{12}}{\sum_{c\subset[15;65]} {Pop}_{c,r}}$

Note that this direct effect is generally quite small due compared to the next type of disruption.^[[5]](#footnote-5)^

Due to the confinement measures used in attempting to internalize the externalities associated with the COVID-19 pandemic, we also allow for the fact that some willing workers become unable to sell their labor because of social distancing policies. We use as a base value the “social-distancing” parameter included in the Imperial College estimates, and assume that 12 weeks of confinement is imposed in each country, except in African countries, for which we limit it to 8 weeks, due to the more limited ability of poor populations to manage long periods of economic disruption; the younger average age of people in the region and the consequent more relaxed implementation of confinement policies. These assumptions result in reductions in the labor supply of 23% in most countries or 15% in Africa. We consider that 1/3 of skilled workers impacted by social distancing can continue working through telecommuting. This crude estimate is based on our review of the ILO’s early review of the impact of Covid-19 on jobs (ILO, 2020) and Dingel and Neiman (2020). Hence, confinement measures lead to an additional reduction of $\beta_{r}^{h}$ for country *r*  and level of skill  *h*, $h\in\left\{ skilled, unskilled \right\}$ such that $\beta_{r}^{h}={Social\_distance}_{r}\times\theta_{r}\times\vartheta_{h}\times\frac{12}{52}$ with $\theta_{r}=2/3$ if *r*  in Sub-Saharan Africa, and 1 otherwise, and $\vartheta_{h}$=$2/3$ if $h="skilled"$ and 1 otherwise.

For the updated scenario, we use the same procedure to link epidemiological projections to the global CGE model, but now using projections from a different epidemiologic model, that is, those of the London School of Hygiene & Tropical Medicine (LHSTM). The projections are described in Pearson et al. (2020). The outcomes of this model provide us with greater detail and flexibility to map individual country projections to actual policy responses. The LSHTM analysis provides ten alternative mitigation scenarios in addition to the unmitigated ones. The process to link these outputs to the economic model through the labor supply restrictions remain the same. The main differences are in how we account for health impacts. These projected impacts, even in the unmitigated scenarios, differ significantly across epidemiologic models, as a result of great disparity in country-specific parameters such as ${Social\_distance}_{r}$, or $\theta_{r}$. The scenario-country mapping is based on a policy-response review,^[[6]](#footnote-6)^ or social-distancing proxy, using Google Mobility reports. Further detail on this mapping can be provided upon request.

Online Appendix A.4. Impacts of COVID-19 for food consumption in China and Nigeria

As noted in Section 5 of the paper, and particularly in Figure 4, the changes in consumption can be considerably sharper at the country level than at the global level. Figure A4 below shows that the dietary shift follows the global pattern in China, but with a markedly stronger increase towards greater consumption of wheat, maize and other grains, while growth of rice—the main staple food—is more modest. The decline in demand for non-staples (fruits and vegetables, animal-sourced products, and vegetable oils) is in line with the global average, albeit slightly stronger. In Nigeria, the picture is more context specific, showing a sharp decline in dairy and vegetable oil consumption along with reductions in demand for certain staple crops, including wheat, as well as sugar and rice. The dietary shift is towards mainly locally produced staples, like maize and other basic grains. In developing countries as a group, consumers shift to maize and other grains while cutting back on vegetables and fruits, fats and oils and dairy products. Even in developed countries, consumption of vegetables and fruits falls substantially.

Figure A4 COVID-19 impacts on diets in China and Nigeria

(Percentage change in household consumption by product)

Source: MIRAGRODEP Simulation (April 2020 scenario).

Note: Results are based on changes in the volume of consumption for each national representative household.

Online Appendix A.5: Summary of Available Results on the Impact of COVID-19

A range of studies summarized in Table A5 has attempted to assess the impacts of COVID-19 on the world economy, on developing economies, and on the poor. Because the magnitude of the economic shocks resulting from COVID-19 was not immediately obvious, many early studies provided much smaller estimates of impact than later reporting studies. Accordingly, the estimates are categorized by whether they were based on the March, June or September quarters. Because most people vulnerable to absolute poverty are in Africa or South Asia, estimates of GDP and poverty impacts are presented for these regions as well as globally.

The IMF World Economic Outlook (WEO) estimates for GDP declines relative to the January 2020 forecast are a useful place to begin. The April WEO suggested a change in global GDP of -6.3% relative to the January forecast, with changes of -5.1% in Africa and -4.1% in India. By June, the global estimate had risen to -8.2%, with a decline of -6.7% in Africa and -10.5% in India. By September, the global estimate had risen slightly, partly because of a more rapid than expected upturn in China, but the outlook for India had deteriorated sharply, with growth of -16.3%. A comparable deterioration in the growth outlook for South Asia and improvement in Africa is evident as we move from the Reference to the October scenarios in this study.

The earliest estimates of the impacts of COVID-19 in Table A5 are those by Maliszewska et al. (2020) and Vos et al. (2020). These studies project declines in global GDP of 2.1% and 3.0% respectively. Based on that modest decline in GDP, Vos et al. (2020) concluded that poverty would rise by 48 million for every 1 percentage point decline in global GDP. This estimate is similar to that of Mahler et al. (2020), who used a much larger estimate of GDP impacts, -6.2%. Part of the difference likely results from the use of uniform income shocks in the Mahler et al study which, as seen in Figure 7 in the main text, is likely to substantially understate the impact of this type of shock on the poor. World Bank (2020a) similarly assumes uniform income shocks, yielding a smaller poverty impact than this study, which differentiates the income shocks and accounts for general equilibrium effects on income distribution.

Table A.5. Estimated Impacts of COVID-19 on GDP and on Poverty

|  |  | **World GDP** | | | **GDP** | **GDP** | **Poverty Headcount** | **Remarks** |
| --- | --- | --- | --- | --- | --- | --- | --- | --- |
|  | **Institution** | **March** | **June** | **Sept** | **Africa** | **S. Asia** | **World** |  |
| This study |  | % | % | % | % | % | millions |  |
| Reference scenario | IFPRI | -5.1 |  |  | -8.9 | -5.0 | 148 | Combined CGE & household models |
| September scenario | IFPRI |  |  | -7.1 | -5.8 | -12.9 | 150 | " |
| Vos et al. (2020) | IFPRI | -3.0 |  |  |  |  | 48 | " |
| WEO April | IMF | -6.3 |  |  | -5.1 | -4.1 |  | Relative to Jan 2020 WEO |
| WEO June | IMF |  | -8.2 |  | -6.7 | -10.5 |  | " |
| WEO Oct | IMF |  |  | -7.7 | -6.5 | -16.3 |  | " |
| Kharas (2020) | Brookings |  |  |  |  |  | 144 | <https://tinyurl.com/y3oyehys> |
| Kharas & Hamel (2020) | Brookings | -6.2 |  |  |  |  | 40-60 | April vs Oct 19 WEO, Poverty Clock |
| Mahler et al (2020) | WB |  | -6.2 |  |  |  | 49 | April vs Oct 19 WEO, Povcalnet |
| Maliszewska et al. (2020) | WB | -2.1 |  |  | -1.4-3.0 | -2.4-5.0 |  | ENVISAGE model |
| McKibbin/Fernando (2020 | ANU |  | -19.4 |  |  |  |  | 17tn of $87.7tn; from estimated impacts |
| OECD | OECD |  | -7.6 |  |  | -7.0 |  | India for South Asia |
| Sumner et al (2020) | WIDER | 5-20 |  |  |  |  | 110-500 | Uses PovcalNet |
| World Bank (2020a) | WB |  |  | -5-8 |  |  | 88-115 | Based on Global Ec Prospects & Povcalnet |
| World Bank (2020b) | WB |  |  | -5-8 |  |  |  | -5 is abs change, -8 change from Jan fcast |
| WTO (2020) | WTO | -2 to -9 |  |  | -4 to -9 | -5 to -13 |  | <https://tinyurl.com/y2zdcpb7> |

**References for Online Appendices**:

Deaton, A. (1989). Rice prices and income distribution in Thailand: A non-parametric analysis. *Economic Journal,* 99 (395), 1–37.

Decreux, Y. & Valin, H. (2007). MIRAGE, updated version of the model for trade policy analysis: Focus on agriculture and dynamics, Working Papers 2007-15, CEPII, Paris <http://cepii.fr/PDF_PUB/wp/2007/wp2007-15.pdf>

Dingel, J. & Neiman, B. (2020). How many jobs can be done from home? White Paper by Becker Friedman Institute, University of Chicago. <https://tinyurl.com/rgt3bmz>

ILO. (2020).  ILO Monitor: COVID-19 and the world of work. Third edition updated estimates and analysis. International Labour Organization. April 29. <https://tinyurl.com/ybhf6n2m>

IMF (2020). *World economic outlook.* International Monetary Fund, Washington DC. https://www.imf.org/en/Publications/WEO

Ivanic, M. & Martin, W. (2018). Sectoral productivity growth and poverty reduction: national and global impacts. *World Development*. 109, 429-439. <https://doi.org/10.1016/j.worlddev.2017.07.004>

Kharas, H. (2020). The Impact of COVID-19 on global extreme poverty, Blog Post, The Brookings Institution, Washington DC. <https://tinyurl.com/y3oyehys>

Kharas, H. & Hamel, K. (2020). Turning back the clock: how will COVID-19 impact the world’s poorest people? Brookings Institution, Washington DC. <https://tinyurl.com/y3exwj5v>

Laborde, D. & Martin, W. (2018). Implications of the global growth slowdown for rural poverty. *Agricultural Economics*, 49(3), 325-338.

Laborde, D., Robichaud, V. & Tokgoz, S. (2013), MIRAGRODEP 1.0: documentation, AGRODEP Technical Note, International Food Policy Research Institute, December.

Mahler, D., Lakner, C, Castaneda Aguilar, R. & Wu, H. (2020). *The impact of COVID-19 (Coronavirus) on global poverty: Why Sub-Saharan Africa might be the region hardest hit*? World Bank Data Blog. April 20**.** <https://tinyurl.com/yalblur8>

Maliszewska, M., Mattoo, A. & van der Mensbrugghe, D. (2020). The potential impact of COVID-19 on GDP and Trade: A preliminary assessment. World Bank Policy Research Working Paper 9211. Washington D.C.: The World Bank.

McKibbin, W. & Fernando, R. (2020). Global macroeconomic scenarios of the COVID-19 pandemic. Centre for Applied Macroeconomic Analysis (CAMA) Working Paper 62/2020. The Australian National University. https://tinyurl.com/y9fafd5t

Muhammad, A., D'Souza, A. Meade, B. Micha, R. & Mozaffarian, D. (2017). The influence of income and prices on global dietary patterns by country, age, and gender. *Economic Research Report* No. ERR-225, US Department of Agriculture. (March). <https://tinyurl.com/y3umwq34>

OECD (2020). *OECD Economic Outlook,* OECD, June.

Pearson, C., Van Zandvoort, K, Jarvis, C., Davies, N., Thompson, S, Checchi, F, Jit, M, Eggo, R., & LSHTM CMMID COVID-19 Working Group (2020). Projections of COVID-19 epidemics in LMIC countries. London School of Hygiene & Tropical Medicine, London, UK. https://doi.org/10.17037/DATA.00001564.

Stone, R. (1954). Linear expenditure systems and demand analysis: an application to the pattern of British demand. *The Economic Journal*, *64*(255), 511-27.

Sumner, A., Hoy, C. & Ortiz-Juarez, E. (2020). Estimates of the impact of COVID-19 on global poverty. WIDER Working Paper 2020/43. UN WIDER.

Vos, R., Laborde, D. & Martin, W. (2020). How much will global poverty increase because of COVID-19?, IFPRI Blog Research Post, https://www.ifpri.org/blog/how-much-will-global-poverty-increase-because-covid-19

Walker, P. et al. (2020). The global impact of COVID-19 and strategies for mitigation and suppression. Imperial College COVID-19 Response Team Report 12. Imperial College. <https://tinyurl.com/ydgglerl>

World Bank (2020a). *Poverty and shared prosperity 2020: reversals of fortune*. World Bank.

World Bank (2020b). *Global Economic Prospects*. World Bank.

WTO (2020). ‘Methodology for the WTO Trade Forecast of April 8 2020, Mimeo, World Trade Organization, Geneva. https://tinyurl.com/y2zdcpb7

1. Details available at https://public.tableau.com/profile/laborde6680#!/vizhome/IFPRI_Blog_Coronavirus_LMV_032020/MainStory (select “model nomenclature”). [↑](#footnote-ref-1)
2. <https://public.tableau.com/profile/laborde6680#!/vizhome/POVANA_Surveys/POVANA> [↑](#footnote-ref-2)
3. *Card(K)* is the cardinal of the set *K*, and therefore *card(K)=211.* For missing countries in FAOSTAT, like the Democratic Republic of Congo, we use a proxy country, in this case, the Central Africa Republic. [↑](#footnote-ref-3)
4. <https://www.worldometers.info/coronavirus/coronavirus-age-sex-demographics/> [↑](#footnote-ref-4)
5. When providing numbers per capita in our results and models, we did not correct the total population used in the counter-factual by the number of deaths. This omission does not affect outcomes in any significant way as the number of deaths relative to the total population is very low. [↑](#footnote-ref-5)
6. See for instance the IFPRI COVID-19 Policy Response Portal ( <https://www.ifpri.org/project/covid-19-policy-response-cpr-portal> ) and the Oxford COVID-19 Government Response Tracker by Hale, Webster, Petherick, Phillips and Kira (2020). [↑](#footnote-ref-6)
